# Clinical Characteristics of Patients With Previous Helicobacter pylori Infection-Induced Atrophic Gastritis

**DOI:** 10.7759/cureus.63368

**Published:** 2024-06-28

**Authors:** Hiroshi Kishikawa, Kenji Nakamura, Sakiko Takarabe, Tadashi Katayama, Aya Sasaki, Soichiro Miura, Yukie Hayashi, Hitomi Hoshi, Takahiro Kanai, Jiro Nishida

**Affiliations:** 1 Gastroenterology, Tokyo Dental College, Ichikawa General Hospital, Chiba, JPN; 2 Clinical Laboratory, Tokyo Dental College, Ichikawa General Hospital, Chiba, JPN; 3 Graduate School, International University of Health and Welfare, Tokyo, JPN; 4 Gastroenterology and Hepatology, Keio University, Tokyo, JPN

**Keywords:** helicobacter pylori, atrophic gastritis, pepsinogen, autoimmune gastritis, gastric cancer screening

## Abstract

Aims: Patients with atrophic gastritis unrelated to autoimmune gastritis (AIG) and without active *Helicobacter pylori *(*H.pylori*) infection or previous eradication therapy are considered to have previous *Helicobacter pylori* infection-induced atrophic gastritis (PHIG). This study aimed to clarify the clinical characteristics of patients with PHIG.

Methods: Consecutive patients who underwent upper gastrointestinal endoscopy during the study period were enrolled in the study. Pepsinogen and gastrin levels,* H. pylori* serology, and endoscopic atrophic grade were assessed. Patients were divided into five groups based on their *H. pylori* status and disease history (PHIG, without *H. pylori* infection, with active *H. pylori* infection, with successful *H. pylori* eradication, and AIG). Their gastric cancer risk status was classified according to the ABC method of serological gastric cancer screening.

Results: Of 536 consecutive patients who underwent upper gastrointestinal endoscopy during the study period, 318 were included (31 with PHIG, 77 without* H. pylori* infection, 101 with active *H. pylori *infection, 80 with successful *H. pylori* eradication, and 29 with AIG). Of the 31 patients with PHIG, 21 (68%) were* H. pylori*-seronegative*,* and 20 (65%) were classified as group A (normal pepsinogen, *H. pylori*-seronegative). Patients with PHIG accounted for 90.1% of the patients at high risk for gastric cancer misclassified as group A. The pepsinogen and *H. pylori* serological profiles of patients with PHIG were similar to those of patients with successful *H. pylori* eradication more than six years previously. A receiver-operating characteristic curve (ROC) analysis that included 13 patients with AIG and without active *H. pylori* infection and no previous eradication therapy and 31 patients with PHIG revealed that an endoscopic atrophy grade of O-III or greater according to the Kimura-Takemoto classification can predict AIG.

Conclusions: Two-thirds of the patients with PHIG were misclassified as being at low risk (group A) according to the ABC method, suggesting that endoscopy is necessary for group A patients. The results of the serological evaluation of PHIG indicated that patients with PHIG may have experienced spontaneous *H. pylori* eradication, possibly because of the use of antibiotics for other conditions. Autoimmune gastritis should be considered in the presence of grade 0-III or greater gastric mucosal atrophy in patients with suspected PHIG, even if the autoantibody and histological findings are not available.

## Introduction

Patients who present with atrophic gastritis (confirmed by endoscopy or histology) and without an active *Helicobacter pylori* (*H. pylori*) infection, previous *H. pylori* eradication therapy, or autoimmune gastritis (AIG) are presumed to have *H. pylori*-induced atrophic gastritis with spontaneous *H. pylori* eradication [1]. This disease state is referred to as previous *H. pylori* infection-induced atrophic gastritis (PHIG) [2]. The diagnostic criteria for PHIG are as follows: absence of *H. pylori* eradication treatment; endoscopic or histological diagnosis of atrophic gastritis; absence of clinically defined AIG; and absence of signs of active *H. pylori* infection [2]. Hiyama et al. [3] reported that some patients who exhibited spontaneous *H. pylori* eradication resembled patients with PHIG; however, patients with AIG were not excluded from their study. To our knowledge, this is the only previous report of unintentional *H. pylori* eradication among outpatients.

Because *H. pylori*-induced chronic atrophic gastritis causes gastric cancer [4], the ABC method is widely used in Japan to assess the risk of gastric cancer [5, 6]. This method classifies patients into four groups based on their pepsinogen level (atrophic or normal) and *H. pylori* serology results (seropositive or seronegative). Patients classified as group B (*H. pylori*-seropositive and normal pepsinogen level), group C (*H. pylori*-seropositive and atrophic pepsinogen level), and group D (H. pylori-seronegative and atrophic pepsinogen level) according to the ABC classification are the main targets of endoscopic monitoring; however, patients classified as group A (*H. pylori*-seronegative and normal pepsinogen level) are considered to be at low risk for gastric cancer [7]. The usefulness of the ABC method has been confirmed by studies performed in several East Asian countries [8, 9] and Finland [10], thus suggesting its validity regardless of ethnicity. However, some patients with gastric atrophy and gastric cancer are classified as group A, possibly because of unintentional *H. pylori* eradication [11]. Kiso et al. [12] reported that high-risk cases of gastric atrophy that resemble PHIG are sometimes misclassified as group A.

Applying a lower cutoff value for the *H. pylori* antibody titer has been suggested as a method of reducing the misclassification of the gastric cancer risk [13-15]; however, some high-risk cases are still misclassified as group A [15]. Moreover, previous studies have reported that approximately one-quarter of patients in group A have signs of atrophy despite having no history of *H. pylori* eradication, suggesting the misclassification of PHIG cases [16, 17]. Although these reports suggest that PHIG-like cases could be misclassified as group A, no study has comprehensively examined the pathogenesis of PHIG, its relationship with pepsinogen levels and *H. pylori* infection, or its relationship with the ABC classification.

We aimed to identify patients with PHIG and characterize their serology and pepsinogen levels to determine the association between PHIG and serological screening methods. We also aimed to gain insights regarding the pathogenesis of PHIG.

## Materials and methods

Participants

A cross-sectional study was conducted using a total of 536 consecutive patients who presented to the gastroenterology outpatient department of the Tokyo Dental College of Ichikawa General Hospital and underwent upper gastrointestinal endoscopy for diagnostic purposes between January 2017 and March 2020 to determine their eligibility for inclusion. A non-probability convenience sampling technique was used to obtain the sample, and all enrolled patients who fulfilled the eligibility criteria described below were included in the analysis. The study was approved by the Research Ethics Committee of the Tokyo Dental College of Ichikawa General Hospital (approval number: I-21-43R). All participants provided written informed consent. Patients with gastric cancer, adenoma, neuroendocrine tumors, or a history of these diseases were excluded. The sample size calculation was determined using a sample size calculator (https://www.calculator.net/sample-size-calculator.html), based on an approximately 15% prevalence of PHIG in the population reported in a previous study [17], with a 95% confidence interval and a 5% margin of error. The minimum sample size required was calculated as 196 patients. The actual sample size analyzed in the study (N = 318) far exceeded this required sample size.

The participants were divided into five groups (Table 1). Autoimmune gastritis was defined as seropositive results for either anti-parietal cell antibodies (APCAs) or anti-intrinsic factor antibodies and pathology consistent with AIG as determined by a biopsy of the gastric body [18]. Endoscopic findings were not included in the definition of AIG. Patients with APCA-negative results without AIG were divided into four groups. A negative urea breath test (13C-UBT) result or stool antigen test (SAT) results after at least eight weeks of eradication treatment was defined as successful *H. pylori* eradication. Patients with an endoscopic atrophy grade of C-I or lower based on the Kimura-Takemoto classification [19], an *H. pylori* antibody titer <10 U/mL, and without prior eradication treatment were defined as those without *H. pylori* infection [20]. Patients with an *H. pylori* antibody titer ≥10 U/mL and without prior eradication therapy, regardless of endoscopic atrophy, were defined as those with an active *H. pylori* infection. Patients with an *H. pylori* antibody titer <10 U/mL and atrophic gastritis grade C-II or higher as determined by endoscopy as well as positive 13C-UBT, SAT, or *H. pylori* culture results were defined as those with an active *H. pylori *infection. Patients with an *H. pylori* antibody titer <10 U/mL and atrophic gastritis grade C-II or higher as determined by endoscopy, as well as negative 13C-UBT and/or SAT results, were classified as having PHIG. Patients with a history of eradication but unknown 13C-UBT or SAT results after eradication and patients with atrophic gastritis (grade C-II or higher) as determined by endoscopy but unknown 13C-UBT or SAT results were excluded because their *H. pylori* infection status was unclear.

**Table 1:** Definition of each subgroup *H. pylori*: *Helicobacter pylori*; AIF: anti-intrinsic factor antibody; AIG: autoimmune gastritis; APCA: anti-parietal cell antibody; SAT: stool antigen test; UBT: urea breath test

Pathophysiology of the analyzed cases	Clinical and serological characteristics
Patients with an active *H. pylori *infection	No prior eradication treatment and 1) *H. pylori *antibody titer ≥10 U/mL or 2) *H. pylori* antibody titer <10 U/mL, atrophic gastritis grade ≥C-II, and positive ^13^C-UBT, SAT, or culture results
Patients with an *H. pylori *eradication	Negative conversion of ^13^C-UBT or SAT results eight weeks after eradication
Patients not infected with *H. pylori *	No prior eradication treatment, atrophic gastritis grade C-I or C-0, and *H. pylori* antibody titer <10 U/mL
Patients with previous *H. pylori* infection-induced atrophic gastritis	No prior eradication treatment and* H. pylori *antibody titer <10 U/mL, atrophic gastritis grade ≥ C-II, and negative ^13^C-UBT, SAT, or culture results
Patients with autoimmune gastritis	Seropositive APCA or AIF results and pathology consistent with AIG

Serum biomarker measurements

Blood was collected after overnight fasting immediately before the endoscopy. Participants’ pepsinogen and gastrin levels, *H. pylori* antibody titers, and APCA levels were tested. Patients with negative APCA results who were suspected of having AIG were also tested to determine the presence of anti-intrinsic factor antibodies. Measurements of pepsinogens, gastrin, and *H. pylori* antibody titers were outsourced to LSI Medience Co., Ltd. (Tokyo, Japan). Measurements of APCAs and anti-intrinsic factor antibodies were performed by H.U. Frontier Co., Ltd. (Tokyo, Japan) [18].

Serum *H. pylori* antibody titers were measured using an enzyme immunoassay kit (E Plate “Eiken” Hp antibody II; Eiken Kagaku Co., Ltd., Tochigi, Japan) according to the manufacturer’s instructions [18]. Patients with an antibody titer <10 U/mL were classified as uninfected in accordance with the cutoff value set by the manufacturer; however, because some previous studies have suggested the utility of lower cutoff values [13,14], a cutoff value <3 U/mL was also used for this study. Patients with 13C-UBT results <2.5% were defined as having negative results according to the manufacturer’s instructions.

Classification using the ABC method

Using the ABC classification recommended by the Japanese Society for Helicobacter Research [11], patients were divided into four groups based on their serum pepsinogen test results (atrophic if the pepsinogen I level was ≤70 ng/mL and the pepsinogen I/II ratio was ≤3; normal if the pepsinogen level did not meet these criteria) and *H. pylori *serology (seronegative if the *H. pylori* immunoglobulin G (IgG) level was <3 U/mL; seropositive if the *H. pylori *IgG level was ≥3 U/mL). The four groups were defined as follows: group A, *H. pylori*-seronegative and normal pepsinogen level; group B, *H. pylori*-seropositive and normal pepsinogen level; group C, *H. pylori*-seropositive and atrophic pepsinogen level; and group D, *H. pylori*-seronegative and atrophic pepsinogen level. Patients with a history of *H. pylori *eradication were excluded from the ABC classification.

Endoscopic examination

All endoscopic procedures were performed using electrical panendoscopes (GIF-HQ290 or GIF-HQ290Z; Olympus Corporation, Tokyo, Japan). The grade of endoscopic atrophy was based on the Kimura-Takemoto classification [19], which classifies endoscopic atrophy into closed types (C-I to C-III), for which the endoscopic atrophic border is recognized on the lesser curvature of the gastric body up to the gastric cardia, and open types (grades O-I to O-III), for which the atrophic border is recognized beyond the cardia and on the greater curvature. We added grade O-IV for severe endoscopic atrophic corpus gastritis, which was defined as vascular visibility extending over the entire greater curvature of the stomach regardless of the antral findings [18]. This category was not included in the original Kimura-Takemoto classification. The Kyoto score [19,20] was also used to assess the presence of atrophic gastritis. Patients with Kimura-Takemoto grades C-0 and C-I with a Kyoto score of 0 were classified as those without* H. pylori *infection with a normal status. Patients with Kimura-Takemoto grades ≥C-II were classified as those with gastritis and an abnormal status. The grade of endoscopic atrophy was assessed by three trained endoscopists (K.N., Y.H., and H.H.) who were blinded to the patients’ clinical backgrounds. The final endoscopic findings were determined by consensus.

Statistical analysis

The Kruskal-Wallis test was used to compare the distribution of continuous variables, including age, serum gastrin, and pepsinogen, among three or more groups. The chi-squared test or Fisher’s exact test was used to compare categorical variables between groups. Subsequently, a residual analysis was performed to evaluate the overall trend of the study population according to sex, age 70 years or older, normal pepsinogen (pepsinogen I level >70 ng/mL or pepsinogen I/II ratio >3), negative *H. pylori* serology (*H. pylori* IgG level <3 U/mL), and hypergastrinemia (gastrin level >150 pg/mL); this analysis specifically focused on cases of PHIG. Patients with PHIG were compared with patients in other groups using the Dunn-Bonferroni post hoc test for variables that were significantly different (p < 0.05) according to the chi-squared test or Fisher’s exact test. The interobserver variability of the three endoscopists was determined using the Fleiss kappa statistic (κ) [21]. Kappa values were interpreted as follows: 0.21 to 0.40, fair agreement; 0.41 to 0.60, moderate agreement; 0.61 to 0.80, substantial agreement; and 0.81 to 1.00, nearly perfect agreement. A receiver-operating characteristic (ROC) analysis and the area under the ROC curve (AUC) were used to determine the optimal cutoff value, sensitivity, and specificity of serological markers and endoscopic atrophic grade. Statistical analyses were performed using IBM SPSS Statistics for Windows (version 25; IBM Corp., Armonk, NY). P < 0.05 was considered statistically significant.

## Results

Baseline characteristics of patients evaluated using the ABC method

**Figure 1:**
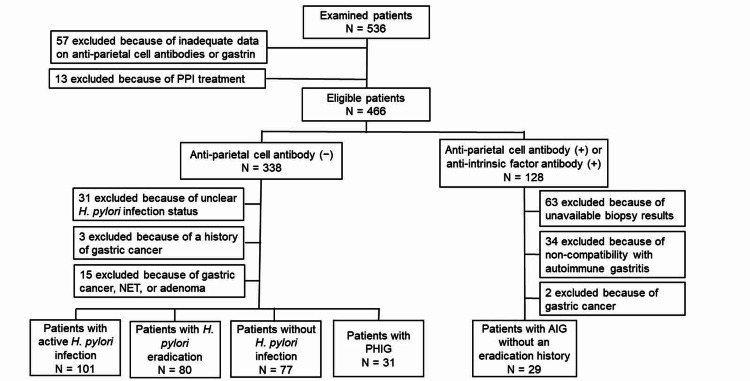
Study flow diagram AIG: autoimmune gastritis; NET: neuroendocrine tumor; PHIG: previous *Helicobacter pylori (H. pylori) *infection-induced atrophic gastritis; PPI: proton pump inhibitor

Of the 536 consecutive patients considered for enrollment, 318 who met the eligibility criteria and had the required clinical and laboratory data were included in the analysis. Of these 318 patients included in the analysis, 31 had PHIG, 101 had an active *H. pylori* infection, 29 had AIG, 80 had successful *H. pylori *eradication, and 77 were without *H. pylori* infection (Figure 1).

Table 2 shows the patients’ baseline characteristics. Patients with PHIG generally had a normal pepsinogen level (94%), negative *H. pylori *serology (68%), were classified as group A according to the ABC method (65%) and did not have severe atrophy according to the Kimura-Takemoto classification (52%).

**Table 2:** Baseline characteristics of the study population IQR: interquartile range; PHIG, previous *Helicobacter pylori* (*H. pylori) *infection-induced atrophic gastritis *Chi-square test or Fisher’s exact test; † Kruskal–Wallis test; ‡Adjusted standardized residual supplemented by a chi-squared test value >1.96; §Adjusted standardized residual supplemented by a chi-squared test value <−1.96

	Clinically confirmed high-risk cases				Clinically confirmed low-risk cases	
	PHIG	Active *H. pylori* infection	Autoimmune gastritis	*H. pylori* successfully eradicated	Normal, no *H. pylori* infection	P-value
	N=31	N=101	N=29	N=80	N=77	
Male, n (%)	14 (45%)	55 (54%)	16 (55%)	34 (43%)^§^	47 (61%)	0.174^*^
Age (years), median (IQR)	70 (66–74)	70 (65–74)	73 (66–75)	72 (68–77)	60 (47.5–71.0)	<0.001^†^
Age older than 70 years, n (%)	17 (55%)	66 (65%)	20 (69%)	55 (69%)^‡^	21 (27 %)^§^	<0.001^*^
Pepsinogen I level (ng/mL), median (IQR)	36.9 (25.2–50.4)	49.9 (30.6–65.4)	6.4 (3.8–17.6)	38.8 (28.2–48.3)	50.4 (39.2–65.9)	<0.001^†^
Pepsinogen II level (ng/mL), median (IQR)	6.3 (5.0–9.4)	14.5 (11.1–22.3)	7.4 (4.9–10.1)	6.1 (4.8–8.1)	7.7 (5.3–10.1)	<0.001^†^
Pepsinogen I/II ratio, median (IQR)	5.9 (4.3–6.7)	3.1 (2.2–4.4)	0.9 (0.6–1.6)	6.1 (5–7.4)	7.0 (6.1–8.5)	<0.001^†^
Normal pepsinogen level, n (%)	29 (94%)^‡^	60 (59%)^§^	2 (7%)^§^	80 (100%)^‡^	75 (97%)^‡^	<0.001^*^
*H. pylori *serology <3 U/mL, n (%)	21 (68%)^‡^	2 (2%)^§^	18 (62%)^‡^	26 (33%)^§^	69 (90%)^‡^	<0.001^*^
ABC classification, n (%)						<0.001†
Group A	20 (65%)	2 (2%)	3 (10%)	26 (33%)	68 (88%)	
Group B	9 (29%)	58 (57%)	1 (3%)	54 (68%)	7 (9%)	
Group C	1 (3%)	41 (41%)	9 (31%)	0 (0%)	1 (1%)	
Group D	1 (3%)	0 (0%)	16 (55%)	0 (0%)	1 (1%)	
Group A, n (%)	20 (65%)^‡^	2 (2%)^§^	3 (10%)^§^	26 (33%)	68 (88%)^‡^	<0.001^*^
Gastrin (pg./mL), median (IQR)	120 (88–136)	132 (96–201.0)	1310 (430–2450)	95 (81–129)	84.0 (70.0–103.0)	<0.001^†^
Hypergastrinemia (>150 pg./mL), n (%)	5 (16%)	40 (40%)^‡^	26 (90%)^‡^	10 (13%)^§^	5 (6%)^§^	<0.001^*^
Endoscopic atrophy grade, n (%)						<0.001†
C-0 or C-I	0 (0%)	12 (12%)	0 (0%)	11 (14%)	77 (100%)	
C-II or C-III	19 (61%)	13 (13%)	2 (7%)	11 (14%)	0 (0%)	
O-I or O-II	8 (26%)	63 (63%)	2 (7%)	48 (60%)	0 (0%)	
O-III or O-IV	4 (13%)	13 (13%)	25 (86%)	10 (13%)	0 (0%)	
Severe endoscopic atrophy (O-I–O-IV), n (%)	12 (39%)^§^	76 (75%)^‡^	27 (93%)^‡^	58 (64%)^‡^	0 (0%)^§^	<0.001^*^

Identification of patients without *H. pylori *infection among patients in group A using the area under the curve

**Figure 2:**
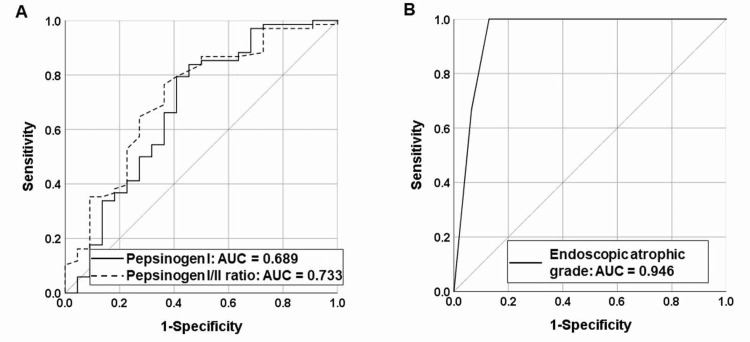
A: Receiver operating characteristic curves for distinguishing H. pylori naïve patients in group A based on pepsinogen values. B: Receiver operating characteristic curves for distinguishing H. pylori-naïve autoimmune gastritis patients from previous H. pylori infection-induced atrophic gastritis (PHIG) patients. AUC: area under the curve

We examined the cutoff value of the pepsinogen level used to identify “true group A” patients without *H. pylori* infection among those serologically classified as group A using the ABC classification. First, of the 318 patients, we identified those who were classified into group A by the ABC classification in daily practice who had no history of eradication, a normal serologic pepsinogen level, and *H. pylori *antibody level <3 U/mL; therefore, a total of 90 patients (68 patients without *H. pylori* infection, 20 with PHIG, and 2 with a current *H. pylori *infection) were classified as group A (Table 3). PHIG accounted for 22% of all cases in group A and 91% of the high-risk cases that were misclassified as group A. The optimal cutoff value that could distinguish the 68 patients without *H. pylori* infection from the other 22 patients (20 with PHIG and 2 with a current *H. pylori* infection) was calculated using the ROC analysis and Youden’s index (Figure 2A). The cutoff values were 38 pg/mL for pepsinogen I (AUC, 0.689; 95% CI, 0.538-0.841) and 6.4 for the pepsinogen I/II ratio (AUC, 0.733; 95% CI, 0.597-0.869).

**Table 3:** Clinical characteristics of patients serologically classified into group A using the ABC method IQR: interquartile range; PHIG: previous *Helicobacter pylori* (*H. pylori) *infection-induced atrophic gastritis

	True group A patients	Misclassified group A patients	
	Patients without an *H. pylori* infection	Patients with PHIG	Patients with an active *H. pylori* infection
	N = 68	N = 20	N = 2
Age (years), median (IQR)	55 (47–70)	68 (47–70)	69
Male (n, %)	43 (63.2%)	8 (40%)	1 (50%)
Endoscopic atrophy grade, n (%)			
C0 or C-I	68 (100%)	0 (0%)	0 (0%)
C-II or C-III	0 (0%)	13 (65%)	1 (50%)
O-I or O-II	0 (0%)	4 (20%)	1 (50%)
O-III or O-IV	0 (0%)	3 (15%)	0 (0%)
Pepsinogen I level (ng/mL), median (IQR)	51.3 (39.5–66.7)	37.2 (26.5–54.2)	47.2
Pepsinogen II level (ng/mL), median (IQR)	7.7 (5.2–9.8)	7 (5.3–9.8)	6.9
Pepsinogen I/II ratio, median (IQR)	7.2 (6.4–8.9)	5.9 (4.1–7.4)	6.9
*H. pylori *serology <3 U/mL, n (%)	68 (100%)	20 (100%)	2 (100%)
Gastrin (pg/mL), median (IQR)	83 (70.5–100.5)	107 (82.3–135.5)	82.5

Comparison of patients with PHIG and patients with successful *H. pylori *eradication

The clinical and serological characteristics of the 31 patients with PHIG and 80 patients with successful *H. pylori *eradication were compared (Table 4) to assess their similarity. Patients with successful eradication were divided into the following three groups according to the time since eradication: <2 years, 27 patients; two to six years, 38 patients; and ≥6 years, 15 patients. All patients with successful *H. pylori* eradication experienced a return to a normal pepsinogen level within two years after eradication therapy, and their normal pepsinogen levels were maintained thereafter. The number of patients with *H. pylori* antibody levels <3 U/mL increased over time after eradication as follows: <2 years, 15%; two to six years, 32%; and ≥6 years, 67%. Therefore, the proportion of group A cases, defined by a negative *H. pylori* antibody and normal pepsinogen levels, also increased over time. The proportion of patients with an *H. pylori* antibody titer <3 U/mL differed significantly between patients with* H. pylori* eradication <2 years previously, eradication two to six years previously, and patients with PHIG. However, patients with PHIG and patients with *H. pylori *eradication ≥6 years previously were serologically similar in terms of the prevalence of an *H. pylori *antibody titer <3 U/mL (68% vs. 67%) and normal pepsinogen level (94% vs. 100%).

**Table 4:** Comparison of patients with previous H. pylori infection-induced atrophic gastritis and those who presented with successful H. pylori eradication PHIG: Previous *Helicobacter pylori* (*H. pylori*) infection-induced atrophic gastritis *Comparison among four groups using the chi-square test or Fisher’s exact test; Difference compared with previous *H. pylori* infection-induced atrophic gastritis cases determined using the Dunn-Bonferroni post hoc test. †p < 0.05; ‡p<0.01.

	Patients with PHIG	Patients who presented with successful* H. pylori* eradication			P-value^*^
Duration after eradication	NA	>6 years	2-6 years	<2 years	
Study participants	N = 31	N = 15	N = 38	N = 27	
Male, n (%)	14 (45%)	10 (67%)	14 (37%)	10 (37%)	0.22
Age older than 70 years, n (%)	15 (48%)	9 (60%)	21 (55%)	18 (67%)	0.56
*H. pylori* serology <3 U/mL, n (%)	21 (68%)	10 (67%)	12 (32%)^†^	4 (15%)^‡^	<0.001
Normal pepsinogen level, n (%)	29 (94%)	15 (100%)	38 (100%)	27 (100%)	0.15
Group A in ABC classification, n (%)	20 (65%)	10 (67%)	12 (32%)^†^	4 (15%)^‡^	<0.001
Hypergastrinemia (>150 pg/mL), n (%)	5 (16%)	3 (20%)	2 (5%)	5 (19%)	0.32

Characteristics of patients without a history of eradication and without evidence of a current *H. pylori *infection but with endoscopic gastric atrophy

Because patients with PHIG and those with *H. pylori*-naïve AIG met the criteria of no evidence of active* H. pylori* infection, no history of* H. pylori *eradication, and gastric mucosa atrophy, an autoantibody test and histological evaluation were required to allow accurate differentiation. However, it would not have been practical to measure the autoantibody levels of all patients with PHIG to discriminate between PHIG and AIG. Therefore, we examined factors that can easily distinguish patients with PHIG from those with AIG. We evaluated 31 patients with PHIG and 15 patients with AIG without a history of *H. pylori* eradication and no current infection. Patients with AIG were significantly less likely than patients with PHIG to have normal pepsinogen levels (p < 0.001), higher gastrin levels (p < 0.001), and more severe atrophy (grade O-III or higher) (p < 0.001) according to the Kimura-Takemoto classification (Table 5). We examined the possibility of differentiating these cases based on endoscopic atrophy using ROC analysis and Youden’s index (Figure 2B). The AUC of the endoscopic atrophic border was 0.95 (95% CI, 0.88-1.0), and the threshold point of the endoscopic atrophic grade used to distinguish patients with PHIG from those with *H. pylori*-naïve AIG was O-III (sensitivity of 87% [27/31] and specificity of 100% (13/13)).

**Table 5:** Characteristics of cases without a history of eradication, without evidence of a current H. pylori infection, and with endoscopic gastric atrophy IQR: interquartile range; PHIG: previous *Helicobacter pylori *(*H. pylori*) infection-induced atrophic gastritis *Chi-square test or Fisher’s exact test; † Kruskal–Wallis test.

	PHIG cases N=31	Autoimmune gastritis without an eradication history and current *H. pylori* infection N=15	P-value*
Male, n (%)	14 (45%)	5 (33%)	0.53*
Age, y, median (IQR)	70 (66-74)	70 (65-77)	0.63†
Age older than 70 years, n (%)	17 (55%)	7/15 (46%)	1*
Pepsinogen I (ng/mL), median (IQR)	36.9 (25.2-50.4)	4.8 (3.8-8.2)	<0.001†
Pepsinogen II (ng/mL), median (IQR)	6.3 (5.0-9.4)	7.4 (4.9-9.2)	0.92†
Pepsinogen I/II ratio, median (IQR)	5.9 (4.3-6.7)	0.8 (0.6-1.1)	<0.001†
Normal pepsinogen, n (%)	29 (94%)	0 (0%)	<0.001*
Anti-parietal cell antibody positive, n (%)	0 (0%)	15 (100%)	<0.001*
*H. pylori *serology <3 U/mL, n (%)	21 (68%)	15 (100%)	1*
				ABC classification, n (%)			
Group A	20 (65%)	0 (0%)	
Group B	9 (29%)	0 (0%)	
Group C	1 (3%)	3 (20 %)	
Group D	1 (3%)	12 (80%)	
Gastrin (pg/mL), median (IQR)	120 (88-136)	2320 (1230-3050)	<0.001†
Hypergastrinemia (>150 pg/mL), n (%)	5 (16%)	15 (100%)	<0.001*
Endoscopic atrophy grade, n (%)			
C-0 or C-I	0 (0%)	0 (0%)	
C-II or C-III	19 (61%)	0 (0%)	
O-I or O-II	8 (26%)	0 (0%)	
O-III or O-IV	4 (13%)	15 (100%)	
Endoscopic atrophy grade >O-III, N (%)	4 (13%)	15 (100%)	<0.001*

Interobserver agreement

The kappa value was used to evaluate the interobserver agreement for classifying endoscopic atrophy as ≤C-I (without atrophy) or ≥C-II (with atrophy). The kappa value was 0.851 (95% CI, 0.777-0.926; p < 0.001), which indicated almost perfect agreement (≥0.81) [21] and confirmed the reliability of the endoscopic diagnosis.

## Discussion

In clinical practice, patients often present with findings of chronic atrophic gastritis despite the absence of an active *H. pylori* infection and a history of *H. pylori *eradication treatment. These include patients with PHIG and some patients with *H. pylori*-naïve AIG. Most cases of PHIG have been speculated to occur in patients after unintentional *H. pylori *eradication attributable to incidental exposure to antibiotics, patients whose *H. pylori* infection is no longer viable and disappears because of progressive atrophy, and patients who may not recall receiving previous *H. pylori* eradication treatment [2]. In the present study, we included patients with PHIG and strictly excluded patients with AIG by measuring the anti-parietal cell antibodies of all eligible patients; therefore, for the first time, we were able to study the clinical features of these patients.

We examined the association between PHIG and serological gastric cancer screening results. The ABC classification is a useful and cost-effective serological method used to perform gastric cancer mass screening [22]. However, several investigators have recognized that some patients at high risk for gastric cancer have false-negative results when the ABC method is used [15,17,23,24]. These false negative cases include active *H. pylori-*infected patients with false negative *H. pylori* serology results, PHIG, and AIG patients. Patients with a history of *H. pylori* eradication were excluded from the ABC classification. During this study, two-thirds of patients with PHIG were included in group A (serologically healthy group), and PHIG accounted for 90% of misclassified cases in group A, suggesting that PHIG is a major source of false negative results of the ABC method. The finding that PHIG is the main cause of the false negative results of the ABC method is consistent with the speculations of previous studies [11,12].

We considered whether it is possible to identify PHIG and other high-risk cases in group A using serology. Recently, two studies have reported serological cutoff values that could be used to differentiate between patients at high risk who were misclassified as group A and patients without *H. pylori* infection who were correctly classified as group A [23,24]. Although the cutoff values for pepsinogen level in two studies were similar (pepsinogen I/II ratios of 4.6 and 5.1), the AUCs were relatively low (0.660 vs. 0.743) for the pepsinogen I/II ratio [23,24]. We re-examined the pepsinogen values of group A with the new ABC classification using a ROC analysis and obtained similar results and relatively low AUCs (0.689 for pepsinogen I and 0.733 for the pepsinogen I/II ratio). Therefore, performing additional serologic testing or changing the cutoff values for pepsinogen would not be useful for reducing the misclassification of cases as group A. The results of this study support the recommendation by the Japanese Society for Helicobacter Research [11] that an endoscopic risk assessment is necessary for group A to exclude patients at high risk. This is an important point that requires the focus of clinicians.

We also attempted to clarify the pathogenesis of PHIG by performing a serological analysis. It has been speculated that most cases of PHIG occur in patients with unintentional eradication of *H. pylori* attributable to accidental exposure to antibiotics; however, this is practically impossible to confirm. Our results showed that the serologic profile of patients with PHIG closely resembles that of patients with successful eradication that occurred more than six years previously. Although the pepsinogen level rapidly normalizes within two months after *H. pylori *eradication therapy [25], *H. pylori *antibody titers decrease gradually, with a reported median time to negative titers of six years [26]. Thus, the results of the present study suggest that many patients with PHIG who were thought to have been *H. pylori*-positive were found to be serologically *H. pylori*-negative over time (probably ≥6 years) after unintentional eradication attributable to the use of antimicrobial agents for the treatment of other conditions. Therefore, it is assumed that most PHIG cases have the same pathogenesis as that observed after *H. pylori* eradication.

Several randomized controlled trials have confirmed that the incidence of gastric cancer decreases after *H. pylori* eradication [27]. However, the risk of gastric cancer for these patients remains higher than that for individuals without a history of *H. pylori *infection, suggesting that patients with PHIG should be followed up with periodic endoscopy, as is performed for patients with eradication.

Although the circumstances under which PHIG occurs have been speculated, it is reasonable to assume that PHIG is caused by a combination of acid secretion inhibitors and antibiotics. Because the increase in intragastric pH caused by proton pump inhibitors is closely related to *H. pylori* disappearance when antimicrobial agents are administered simultaneously, the simultaneous administration of proton pump inhibitors and antibiotics for reasons other than *H. pylori *eradication could theoretically induce spontaneous (unintentional) *H. pylori *eradication. The frequent use of antibiotics and proton pump inhibitors [28] could contribute to an increased incidence of PHIG.

*Helicobacter pylori*-naïve AIG is an autoimmune disease involving gastritis that is not caused by *H. pylori* infection. Thus, patients with PHIG and some patients with AIG have no history of *H. pylori* eradication, no evidence of active *H. pylori *infection, and atrophy of the gastric mucosa. Half of the patients with AIG included in this study met these criteria and were considered as those with *H. pylori*-naïve AIG. A strict diagnosis of AIG requires pathological findings and autoantibodies; however, it is impractical to use all of these for all cases during clinical trials. However, the ROC analysis revealed that AIG can be effectively differentiated from PHIG by endoscopic findings indicating grades O-III or higher, which are clinically useful indicators. During future clinical studies, PHIG might be predicted based on endoscopic findings. Thus, another important point that requires the attention of clinicians is that AIG should be considered even in the presence of severe gastric mucosal atrophy with grade O-III or higher, even if the clinical *H. pylori *profile and gastric atrophy are similar to those of PHIG. This may allow the discovery of AIG, which is often overlooked.

This study had some limitations. First, it included patients who underwent upper gastrointestinal endoscopy at a single institution. Therefore, the generalizability of the results may be limited. For example, the proportion of patients with AIG in the study population was considerably higher than that in real-world practice. Patients with APCA-negative AIG were not distinguishable from patients without AIG; however, considering the low incidence of AIG in the general population [29] and the relatively high sensitivity of APCA [30], the effect of this misclassification was likely negligible.

## Conclusions

We identified the serological and clinical characteristics of patients with strictly defined PHIG. This study confirmed that patients with PHIG are prone to false negative *H. pylori* serology results and serological risk classifications. These results indicated the limitations of serological methods and that an endoscopic risk assessment using the ABC method is necessary for patients in group A to exclude those with false negative results indicating PHIG. A comparison of the serology of patients with PHIG and patients with successful *H. pylori* eradication showed striking similarities, suggesting that many patients with PHIG are likely to have experienced unintentional *H. pylori* eradication after the use of antimicrobials for other diseases. Clinically, AIG should be considered in the presence of severe gastric mucosal atrophy, even if the autoantibody (including APCAs and anti-intrinsic factor antibodies) and histology results are not available but are clinically similar to those of PHIG. Finally, because PHIG is a common condition in clinical practice that may be increasingly encountered in the future, patients with PHIG should be distinguished from those who are *H. pylori*-positive, those who are uninfected, and those with eradicated cases in clinical trials. Furthermore, it is necessary to evaluate the risk of carcinogenesis and endoscopic intervals for patients with PHIG.

## References

[1] Kokkola A, Kosunen TU, Puolakkainen P (2003). Spontaneous disappearance of Helicobacter pylori antibodies in patients with advanced atrophic corpus gastritis. APMIS.

[2] Kishikawa H, Ojiro K, Nakamura K (2020). Previous Helicobacter pylori infection-induced atrophic gastritis: a distinct disease entity in an understudied population without a history of eradication. Helicobacter.

[3] Hiyama T, Quach DT, Le QD (2015). Rate of unintended Helicobacter pylori eradication in the Vietnamese. Helicobacter.

[4] Correa P (1992). Human gastric carcinogenesis: a multistep and multifactorial process-First American Cancer Society Award Lecture on Cancer Epidemiology and Prevention. Cancer Res.

[5] Miki K (2011). Gastric cancer screening by combined assay for serum anti-Helicobacter pylori IgG antibody and serum pepsinogen levels - "ABC method". Proc Jpn Acad Ser B Phys Biol Sci.

[6] Takahashi Y, Yamamichi N, Kubota D (2022). Risk factors for gastric cancer in Japan in the 2010s: a large, long-term observational study. Gastric Cancer.

[7] Watabe H, Mitsushima T, Yamaji Y (2005). Predicting the development of gastric cancer from combining Helicobacter pylori antibodies and serum pepsinogen status: a prospective endoscopic cohort study. Gut.

[8] Kwak MS, Chung GE, Chung SJ, Kang SJ, Yang JI, Kim JS (2018). Predicting the development of gastric neoplasia in a healthcare cohort by combining Helicobacter pylori antibodies and serum pepsinogen: a 5-year longitudinal study. Gastroenterol Res Pract.

[9] Chen XZ, Huang CZ, Hu WX, Liu Y, Yao XQ (2018). Gastric cancer screening by combined determination of serum Helicobacter pylori antibody and pepsinogen concentrations: ABC method for gastric cancer screening. Chin Med J (Engl).

[10] Song M, Camargo MC, Weinstein SJ (2018). Serum pepsinogen 1 and anti-Helicobacter pylori IgG antibodies as predictors of gastric cancer risk in Finnish males. Aliment Pharmacol Ther.

[11] (2023). The Japanese Society for Helicobacter research: reminder regarding the “serum Helicobacter pylori antibody test” result determination (in Japanese). https://www.jshr.jp/medical/committee/file/past/20150630_igg.pdf.

[12] Kiso M, Yoshihara M, Ito M (2017). Characteristics of gastric cancer in negative test of serum anti-Helicobacter pylori antibody and pepsinogen test: a multicenter study. Gastric Cancer.

[13] Kishikawa H, Kimura K, Ito A (2015). Predictors of gastric neoplasia in cases negative for Helicobacter pylori antibody and with normal pepsinogen. Anticancer Res.

[14] Inoue M, Sawada N, Goto A, Shimazu T, Yamaji T, Iwasaki M, Tsugane S (2020). High-negative anti-Helicobacter pylori IgG antibody titers and long-term risk of gastric cancer: results from a large-scale population-based cohort study in Japan. Cancer Epidemiol Biomarkers Prev.

[15] Kotachi T, Ito M, Yoshihara M (2017). Serological evaluation of gastric cancer risk based on pepsinogen and Helicobacter pylori antibody: relationship to endoscopic findings. Digestion.

[16] Nagasaki N, Ito M, Boda T, Kotachi T, Takigawa H, Oka S, Tanaka S (2022). Identification of Helicobacter pylori-related gastric cancer risk using serological gastritis markers and endoscopic findings: a large-scale retrospective cohort study. BMC Gastroenterol.

[17] Kishino T, Oyama T, Tomori A, Takahashi A, Shinohara T (2020). Usefulness and limitations of a serum screening system to predict the risk of gastric cancer. Intern Med.

[18] Kishikawa H, Nakamura K, Ojiro K (2022). Relevance of pepsinogen, gastrin, and endoscopic atrophy in the diagnosis of autoimmune gastritis. Sci Rep.

[19] Kimura K, Takemoto T (1969). An endoscopic recognition of the atrophic border and its significance in chronic gastritis. Endoscopy.

[20] Toyoshima O, Nishizawa T (2022). Kyoto classification of gastritis: advances and future perspectives in endoscopic diagnosis of gastritis. World J Gastroenterol.

[21] Fleiss JL (1971). Measuring nominal scale agreement among several raters. Psychol Bull.

[22] Saito S, Azumi M, Muneoka Y (2018). Cost-effectiveness of combined serum anti-Helicobacter pylori IgG antibody and serum pepsinogen concentrations for screening for gastric cancer risk in Japan. Eur J Health Econ.

[23] Chinda D, Shimoyama T, Mikami T (2018). Serum pepsinogen levels indicate the requirement of upper gastrointestinal endoscopy among group A subjects of ABC classification: a multicenter study. J Gastroenterol.

[24] Kishikawa H, Kimura K, Ito A (2017). Cutoff pepsinogen level for predicting unintendedly eradicated cases of Helicobacter pylori infection in subjects with seemingly normal pepsinogen levels. Digestion.

[25] Kawai T, Miki K, Ichinose M (2007). Changes in evaluation of the pepsinogen test result following Helicobacter pylori eradication therapy in Japan. Inflammopharmacology.

[26] Tanaka S, Goto A, Yamagishi K (2023). Long-term response of Helicobacter pylori antibody titer after eradication treatment in middle-aged Japanese: JPHC-NEXT Study. J Epidemiol.

[27] Ford AC, Yuan Y, Moayyedi P (2020). Helicobacter pylori eradication therapy to prevent gastric cancer: systematic review and meta-analysis. Gut.

[28] Luo H, Fan Q, Xiao S, Chen K (2018). Changes in proton pump inhibitor prescribing trend over the past decade and pharmacists' effect on prescribing practice at a tertiary hospital. BMC Health Serv Res.

[29] Lenti MV, Rugge M, Lahner E (2020). Autoimmune gastritis. Nat Rev Dis Primers.

[30] Lahner E, Norman GL, Severi C (2009). Reassessment of intrinsic factor and parietal cell autoantibodies in atrophic gastritis with respect to cobalamin deficiency. Am J Gastroenterol.

